# Design of a multi-epitope-based vaccine candidate against Bovine Genital Campylobacteriosis using a reverse vaccinology approach

**DOI:** 10.1186/s12917-024-04006-x

**Published:** 2024-04-19

**Authors:** Marta Filipa Silva, Gonçalo Pereira, Luísa Mateus, Luís Lopes da Costa, Elisabete Silva

**Affiliations:** 1 CIISA – Centre for Interdisciplinary Research in Animal Health, Faculty of Veterinary Medicine, University of Lisbon, Lisbon, Portugal; 2 Associate Laboratory for Animal and Veterinary Science (AL4AnimalS), Lisbon, Portugal; 3 Faculty of Veterinary Medicine, Lusófona University, Lisbon, Portugal

**Keywords:** Bovine genital campylobacteriosis, *Campylobacter fetus* subsp. *venerealis*, Reverse vaccinology, Vaccine

## Abstract

**Background:**

Bovine Genital Campylobacteriosis (BGC), a worldwide distributed venereal disease caused by *Campylobacter fetus* subsp. *venerealis* (*Cfv*), has a relevant negative economic impact in cattle herds. The control of BGC is hampered by the inexistence of globally available effective vaccines. The present *in silico* study aimed to develop a multi-epitope vaccine candidate against *Cfv* through reverse vaccinology.

**Results:**

The analysis of *Cfv* strain NCTC 10354 proteome allowed the identification of 9 proteins suitable for vaccine development. From these, an outer membrane protein, OmpA, and a flagellar protein, FliK, were selected for prediction of B-cell and T-cell epitopes. The top-ranked epitopes conservancy was assessed in 31 *Cfv* strains. The selected epitopes were integrated to form a multi-epitope fragment of 241 amino acids, which included 2 epitopes from OmpA and 13 epitopes from FliK linked by GPGPG linkers and connected to the cholera toxin subunit B by an EAAAK linker. The vaccine candidate was predicted to be antigenic, non-toxic, non-allergenic, and soluble upon overexpression. The protein structure was predicted and optimized, and the sequence was successfully cloned *in silico* into a plasmid vector. Additionally, immunological simulations demonstrated the vaccine candidate’s ability to stimulate an immune response.

**Conclusions:**

This study developed a novel vaccine candidate suitable for further in vitro and in vivo experimental validation, which may become a useful tool for the control of BGC.

**Supplementary Information:**

The online version contains supplementary material available at 10.1186/s12917-024-04006-x.

## Background

Bovine Genital Campylobacteriosis (BGC) is a venereal disease of cattle with worldwide distribution caused by *Campylobacter fetus* subsp. *venerealis (Cfv)* [1, 2]. Infected bulls become life-long carriers of *Cfv*, asymptomatically carrying the pathogen in the prepuce, penis and semen, and spreading it through natural breeding or artificial insemination [3, 4]. In females, *Cfv* causes embryonic and early foetal death and abortion, but infection is usually self-limiting and cleared after establishment of an effective immune response, although *Cfv* may persist for long periods in the genital tract [4, 5]. This infertility pattern is responsible for significant economic losses, namely in beef herds [6, 7]. In fact, it is estimated that BGC lead to up to 66% reduction in gross profit margins in the first year of infection [8].

The control of BGC is challenging and expensive, encompassing antibiotic treatment or culling of infected bulls, artificial insemination, and vaccination [8, 9], the latter being regarded both as a prophylactic and a potential therapeutic strategy [4]. Several studies evaluated the efficacy of vaccines as a therapy for BGC infected cows and bulls [10–13]. Although most studies focused on bacterin vaccines [11–13], *C. fetus* cellular extracts were also tested [10]. However, there is a lack of evidence about the efficacy of vaccines against BGC [7, 13], which are not commercially available in many geographical regions, including Europe [14]. A study of Fóscolo et al. [12] demonstrated that the administration of two doses of *Cfv* bacterin to infected bulls decreased the number of positive animals, which is in accordance with previous findings of Vasquez et al. [11]. However, a more recent study [13] found no efficacy on the use of a commercial monovalent vaccine, combined with oxytetracycline treatment, in infected bulls. Similarly, in heifers bred with infected bulls, two commercial vaccines revealed no significant efficacy [15]. Vaccine failures have been related to surface antigenic variation of *Cfv* or the existence of diversity in surface antigens among regional strains [15].

Vaccines against pathogens evolved from incorporating whole organisms (e.g. bacterins) to recombinant vaccines, including multi-epitope vaccines, which are composed of highly antigenic peptides capable of eliciting an effective immune response [16]. Whereas the traditional methods for vaccine development are expensive and time-consuming, the recently emerged reverse vaccinology technology and bioinformatic tools have significantly reduced the time and cost of vaccine development [17]. A new generation of vaccines arose from these novel strategies based on analysis of pathogen’s genome and identification of proteins with favourable characteristics to be used as vaccine targets [18]. In fact, several vaccines with promising results have been developed against other *Campylobacter* species, using reverse vaccinology approaches [19, 20]. However, to the best of our knowledge, this approach was not described to develop a vaccine against *Cfv*. Therefore, this study aimed to identify potential vaccine targets and design a multi-epitope vaccine against *Cfv* for the control of BGC, using a reverse vaccinology approach.

## Results

### Protein analysis and prioritization

**Fig. 1:**
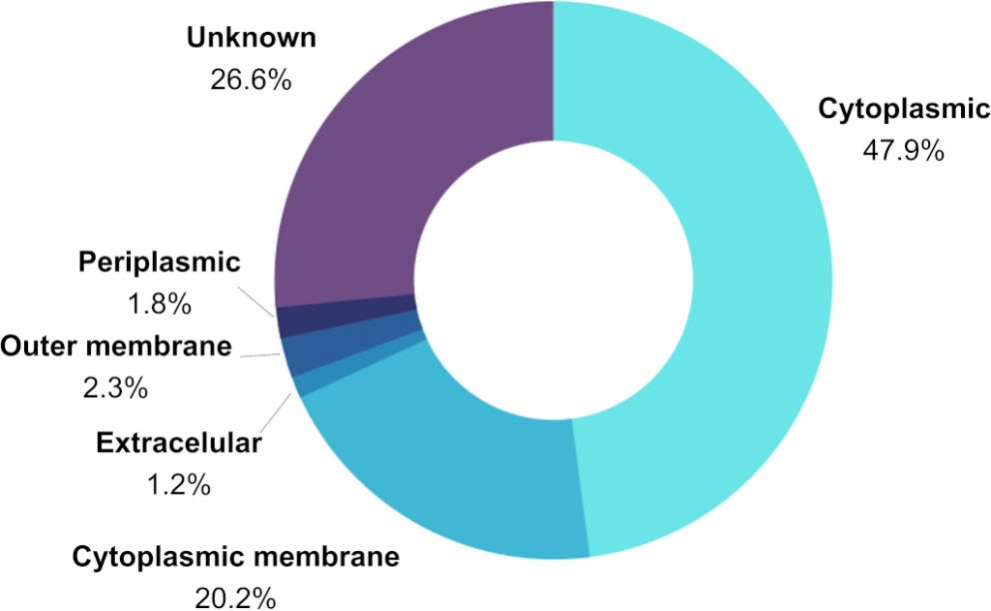
Subcellular localization of *C. fetus* subsp. *venerealis* proteins, predicted using PSORTb version 3.0.3

A total of 1849 proteins of *Cfv* NCTC 10354 were analysed as potential vaccine targets. The subcellular localization, predicted with PSORTb software (version 3.0.3), revealed 23 extracellular and 43 outer membrane proteins (Fig. 1). Using VFanalyzer, 21 of these proteins were identified as potential virulence factors of *Campylobacter* sp. (Table 1).

**Table 1:** Potential virulence factors of *C. fetus* subsp. *venerealis* with extracellular and outer membrane location

Protein ID	Annotated protein	VFDB related virulence factor (gene)
WP_035169143.1	Flagellar hook-basal body complex protein	Flagella (*flgE*)
WP_011731675.1	Flagellar hook-length control protein FliK	Flagella (undetermined)
WP_002847942.1	Cytolethal distending toxin nuclease subunit Cf-CdtB	CDT (*cdtB*)
WP_011731693.1	Flagellar filament capping protein FliD	Flagella (*fliD*)
WP_002848088.1	Flagellar hook-associated protein FlgK	Flagella (*flgK*)
WP_002848571.1	OmpA family protein	CadF (*cadF*)
WP_002848586.1	Major outer membrane protein	MOMP (*porA*)
WP_149120580.1	Cell surface protein	SapA-like
WP_149120581.1	S-layer protein	SapA-like
WP_149120582.1	Cell surface protein	SapA-like
WP_149120584.1	S-layer protein	SapA
WP_080947443.1	Cell surface protein	sapA-like
WP_002848865.1	Flagellar basal-body rod protein FlgG	Flagella (*flgG*)
WP_002849083.1	Flagellar hook protein	Flagella (*flgL*)
WP_002849268.1	Flagellar basal body L-ring protein FlgH	Flagella (*flgH*)
WP_002849489.1	Cytolethal distending toxin subunit B family protein	CDT (*cdtB*)
WP_002849498.1	Cytolethal distending toxin subunit B family protein	CDT (*cdtB*)
WP_024305174.1	Transporter substrate-binding domain-containing protein	PEB1/CBF1 (*pebA*)
WP_002850708.1	Flagellin B	Flagella (*flaA*)
WP_002850711.1	Flagellin B	Flagella (*flaB*)
WP_024305360.1	Flagellar hook protein FlgE	Flagella (*flgE2*)

VFDB – Virulence factor database

Among these potential virulence factors, 5 Sap and Sap-like proteins (WP_149120584.1, WP_080947443.1, WP_149120581.1, WP_149120580.1, WP_149120582.1) were excluded due to the high-frequency of antigenic variation of surface layer proteins (SLPs). The remaining 16 proteins were evaluated for antigenicity and non-allergenicity and 5 of them (WP_024305174.1, WP_002847942.1, WP_002849083.1, WP_002849489.1 and WP_002848586.1), predicted as allergens, were excluded.

In order to avoid cross-reactivity of the vaccine with bovine proteins, the selected 11 proteins were subjected to Position-Specific Iterated BLAST (PSI-BLAST) against the *Bos taurus* proteome, and none of them revealed hits, a feature required to be kept to remain in the candidate list. With exception of WP_002849498.1, all proteins fulfilled the required physicochemical inclusion criteria. Protein WP_002849498.1 was excluded because revealed a 45.34 instability index and a 0.094 grand average of hydropathicity (GRAVY), which denotes a lack of stability and of hydrophilic properties. Protein WP_002850708.1 was also excluded for having 2 transmembrane helices, which would hamper the protein purification process.

The final list of candidates included 8 flagellar proteins and one outer membrane protein (Table 2). From these, 2 vaccine candidates were selected based on their cellular role and antigenicity. The Flagellar hook-length control protein FliK (WP_011731675.1) was selected from the flagellar proteins due to the high antigenicity score and the important role in flagellum assembly [21]. The Outer membrane protein A (ompA) family (WP_002848571.1) was selected due to the different cell localization and function. Thereafter, both proteins were subjected to B and T-cell epitope analysis to design a multi-epitope vaccine.

**Table 2:** Shortlist of *C. fetus* subsp. *venerealis* proteins with appropriate characteristics for vaccine development

Protein ID	Annotated protein	VaxiJen score
WP_035169143.1	Flagellar hook-basal body complex protein	0.6452
WP_011731675.1	Flagellar hook-length control protein FliK	0.8215
WP_011731693.1	Flagellar filament capping protein FliD	0.6979
WP_002848088.1	Flagellar hook-associated protein FlgK	0.6033
WP_002848571.1	OmpA family protein	0.6597
WP_002848865.1	Flagellar basal-body rod protein FlgG	0.5069
WP_002849268.1	Flagellar basal body L-ring protein FlgH	0.6385
WP_002850711.1	Flagellin B	0.7581
WP_024305360.1	Flagellar hook protein FlgE	0.7152

### Epitope prediction and analysis

Linear B-cell epitope prediction using BepiPred 2.0 identified 7 peptide sequences for FliK and 8 peptide sequences for OmpA, with more than 10 residues above the threshold, as shown in Table 3.

**Table 3:** Predicted linear B-cell epitope sequences

Protein ID	Start position	Predicted epitope
WP_011731675.1	7	LLNNTASSTMQTKPSDSHESSNDSF
	37	NSVNKNENISEESSKSVVEQNAKKSQIDKKDEKIDSKSNPDEIETEENSSQESPNKNSISILEDA
	115	DGKKLDKFPALSN
	134	STEKNINEIKGAKS
	180	KALEAKGFFKIQDTQIVLNEVTKQKIEKSLKIDKKSDESTPSLTKLLQSTQTIDDASGKKTKNKTETNNIKEATEDTINLKNTDKSKISVEQAEQKNSKKADLNGEKNAIQNTKNIETELTKNDKHTKDTSGATANHTSQKNAKDTNVDDYLANIMQRAIKESSETKDKQSQTTLSGSFTKETKNSGEKDNMQNNSDQNGSQTSSNQSVKDVVANSKLQLKNGQIKQTFES
	416	QEKIAEYKPPVTRFHMTLNPTNLGE
	468	QNQAEFKNSLVNMGFTELSMNFSDQNKNKEQGQNSKFKNYDNDFENVLNQNEDEQVI
WP_002848571.1	32	HPEGTQGIDDQNF
	64	GFDYSKNVSFEHKNGVPVGFET
	114	YQDFTREANDVEDG
	145	ALKAEARDALDWNNGSNTFLY
	168	GFAVGFGESRSAAPAPVLVEEQPAPAPTKIVPAIGDEDGDGVLDNVDRCPGTPKGVVVDEYGCE
	241	NFAFDSAKVTPEYE
	280	STGPEDYNKKLSVK
	317	FGEEQPIASNATKEGRAEN

These sequences of linear B-cell epitopes were used for prediction of peptide sequences binding to MHC class I and class II alleles. For MHC class I binding, FliK (WP_011731675.1) revealed 75 epitopes, whereas OmpA (WP_002848571.1) revealed 19. However, only 33 FliK and 3 OmpA epitopes were predicted as antigens, non-allergenic, non-toxic and water-soluble (Additional file 1). From this shortlist, the epitopes with potential to bind to a higher number of MHC alleles and with higher antigenicity scores were chosen to be included in the multi-epitope vaccine design. Finally, to avoid nucleotide sequence overlaps, the vaccine candidate included 8 epitopes from FliK and 1 from OmpA binding to MHC class I molecules. From MHC class II binding, FliK revealed 27 epitopes and OmpA 2 epitopes. After applying the same criteria described above, 5 FliK and 1 OmpA epitopes were chosen for vaccine design. A BLAST analysis revealed conservancy of all the selected epitopes in a collection of 31 *Cfv* genomes.

### Multi-epitope vaccine design and evaluation

**Fig. 2:**
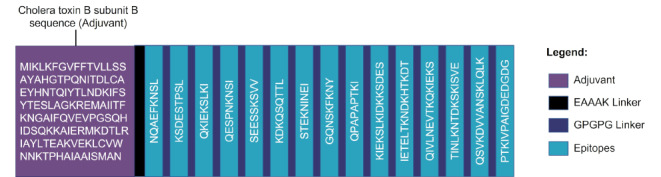
Structural arrangement of the multi-epitope vaccine candidate

The vaccine design included an adjuvant to enhance the immunogenicity of the vaccine construct and 15 epitopes of FliK and OmpA (Table 4). The epitopes were linked together through GPGPG linkers and the multi-epitope fragment was combined with the cholera toxin B subunit sequence as adjuvant, using an EAAAK linker (Fig. 2).

**Table 4:** T-cell epitopes predicted from B-cell epitope sequences included in the vaccine candidate design

MHC class	Epitope Sequence	Protein Source
MHC class I binding epitopes	NQAEFKNSL	FliK
	KSDESTPSL	
	QKIEKSLKI	
	QESPNKNSI	
	GQNSKFKNY	
	SEESSKSVV	
	KDKQSQTTL	
	STEKNINEI	
	QPAPAPTKI	OmpA
MHC class II binding epitopes	QSVKDVVANSKLQLK	FliK
	KIEKSLKIDKKSDES	
	IETELTKNDKHTKDT	
	QIVLNEVTKQKIEKS	
	TINLKNTDKSKISVE	
	PTKIVPAIGDEDGDG	OmpA

The vaccine construct is composed of 370 amino acids, with a molecular weight of 38550.51 and a theoretical isoelectric point (pI) of 9.05. This construct is considered stable, with a predicted instability index of 33.23, and an aliphatic index and GRAVY of 65.92 and − 0.701, respectively, reflecting its thermal stability and hydrophilic nature. The estimated half-life of the vaccine candidate is 30 h in mammalian reticulocytes (in vitro), more than 20 h in yeast (in vivo) and more than 10 h in *Escherichia coli* (in vivo). Subsequent testing revealed that the vaccine was highly antigenic (VaxiJen antigenicity score: 0.9891), non-allergenic and non-toxic.

**Fig. 3:**
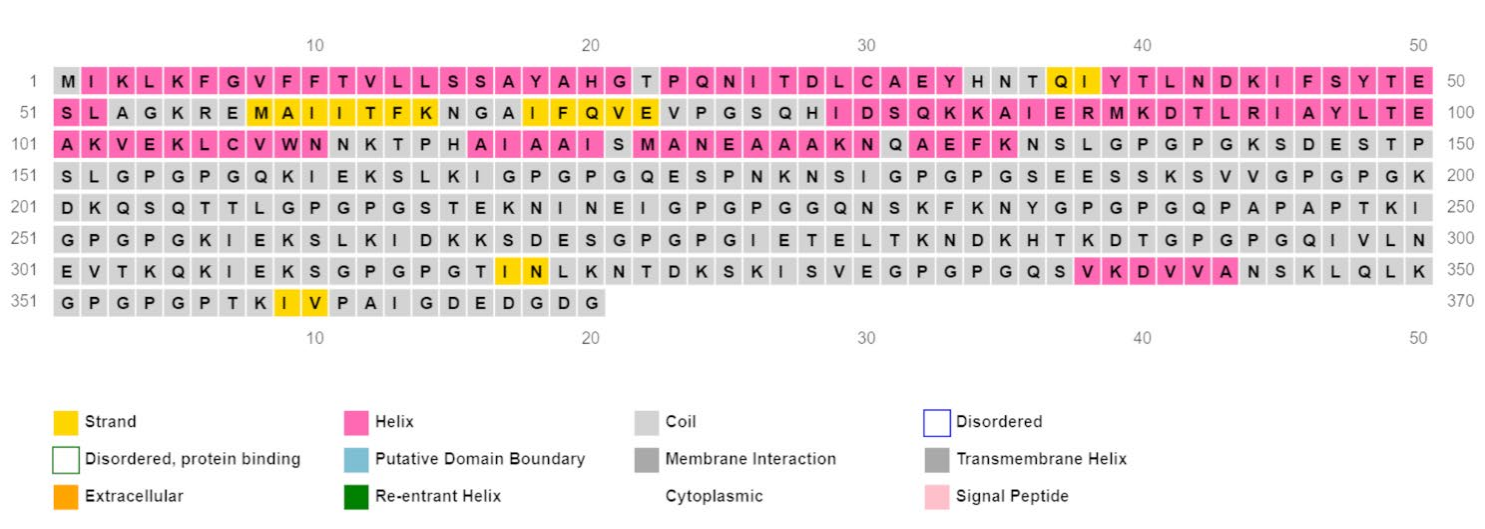
Predicted secondary structure of the vaccine construct by PSIPRED 4.0 server

**Fig. 4:**
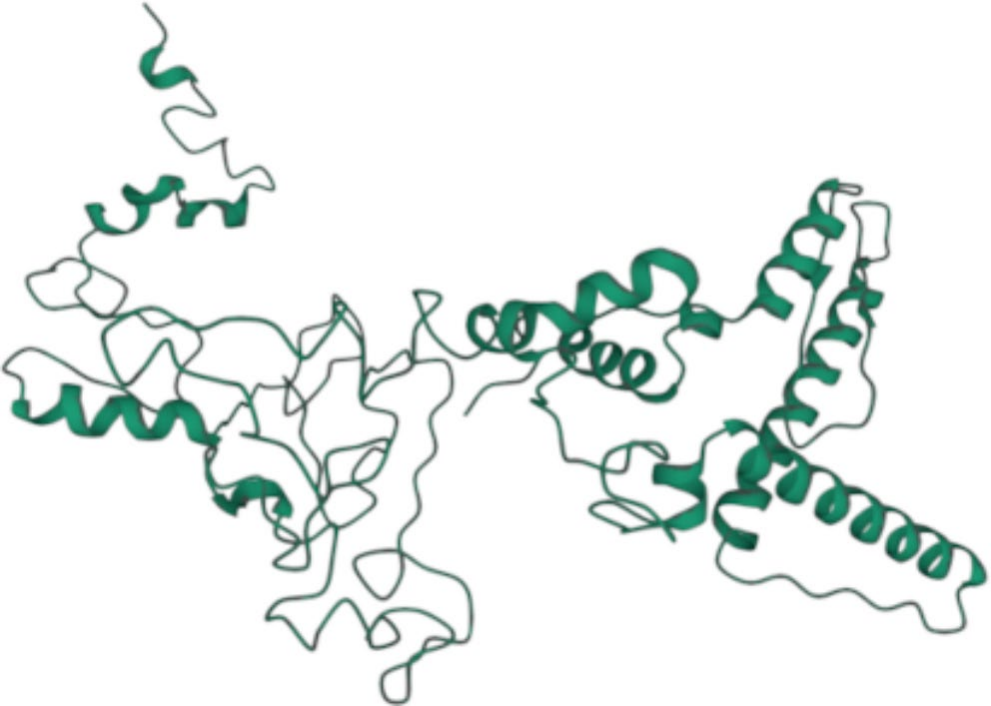
Predicted tertiary structure of the vaccine construct by Scratch Protein Predictor – 3Dpro

The secondary structure of the 370 amino acid sequence was predicted using PSIPRED 4.0, which showed the distribution of amino acids in helices (27.3%), coils (67.8%) and strands (4.9%) (Fig. 3). The tertiary structure was predicted with Scratch Protein Predictor – 3Dpro and visualized with Mol* 3D Viewer, as shown in Fig. 4. This structure was then refined with the Galaxy Refine tool, generating 5 refined models (Table 5). From these, Model 1 was chosen due to an improved MolProbity score (1.991), a lower clash score (13.4), a low percentage of poor rotamers (0.3) and a high-percentage of Ramachandran favored residues (94.8).

**Table 5:** Properties of the refined models

Model	GDT-HA	RMSD	MolProbity	Clash score	Poor rotamers	Rama favored
Initial	1.0000	0.000	3.764	114.2	7.1	88.0
MODEL 1	0.9047	0.542	1.991	13.4	0.3	94.8
MODEL 2	0.9081	0.527	2.007	13.9	0.0	94.8
MODEL 3	0.9074	0.512	2.048	14.8	0.7	94.6
MODEL 4	0.8946	0.555	2.179	16.8	1.3	94.8
MODEL 5	0.9000	0.530	2.093	15.4	1.0	94.0

To improve protein structure stability, Model 1 was subjected to disulfide engineering using Disulfide by Design 2.0. The analysis showed 32 pairs of residues with potential to be used in disulfide engineering. From these, 6 residues were selected to mutate to cysteine for having an energy value less than 2.2 kcal/mol (Additional file 2).

### Codon optimization and in silico cloning

Codon optimization was conducted using the Java Codon Adaptation Tool to achieve optimal expression in *Escherichia coli* K12 strain. The improved cDNA sequence with a length of 1110 bp revealed a GC content of 50.36% and a codon adaptation index (CAI) score of 1.0, suggesting a high expression level. A stop codon (TAA) was added to the optimized codon sequence at the 3’ end to ensure termination of gene translation. The restriction sites for NotI and BamHI enzymes were added to 3´ and 5´ends, respectively, to enable *in silico* cloning into the pET-30a(+) expression vector (Additional file 3: Figure S1). The insertion of the vaccine nucleotide sequence into the expression vector resulted in a plasmid with a length of 6510 bp.

### Immune simulation

**Fig. 5:**
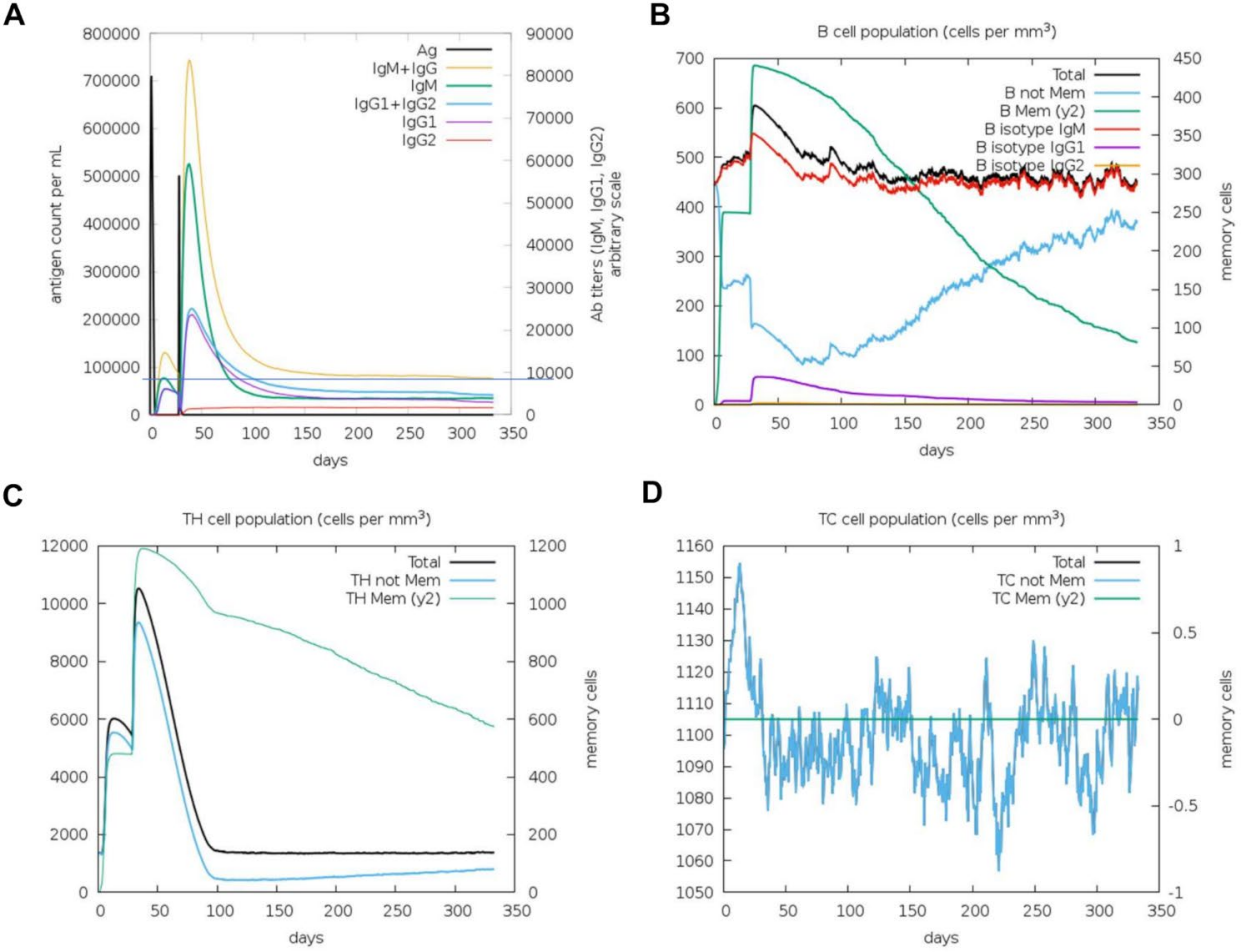
Immune simulation performed by the C-ImmSim server. Prediction of immunoglobulin production in response to antigen administrations (**A**), and evolution of B-cell (**B**), T-helper cell (**C**) and T-cytotoxic cell (**D**) populations

The immune simulation performed with C-ImmSim server showed the ability of the vaccine candidate to induce an immune response through an increase in B and T-cell populations and immunoglobulin titers (Fig. 5).

The levels of IgM and IgG antibodies increased after administration of the vaccine candidate, peaking to IgM + IgG titers over 80,000 after a second administration at day 28 (Fig. 5-A). Although the antibody levels decreased over time, lower levels of IgG and IgM (IgG + IgM titers around 10,000) persisted during the entire simulation period. The vaccine stimulated an increase in the B-cell population (Fig. 5-B), attaining the higher levels after the second vaccine administration. The number of memory B-cells peaked at approximately 450 cells per mm^3^ after the second vaccine administration and, over the course of the simulation period, this number gradually declined to around 100 cells per mm^3^. Likewise, the immune simulation showed that the vaccine administration resulted in an increase in T-helper and T-cytotoxic cell populations (Fig. 5-C, D).

## Discussion

This study used reverse vaccinology methodology to design a multi-epitope vaccine candidate against *Cfv.* The analysis of the subcellular localization of *Cfv* proteins revealed 23 extracellular and 43 outer membrane proteins. These surface-exposed proteins are particularly promising vaccine targets, as they may contribute to the pathogen’s virulence and are exposed to the host immune system [17]. The prediction of their virulence potential allowed the identification of candidates that may play a role in disease pathogenesis, and are susceptible to being neutralized through an antibody-mediated immune response [22]. The final list of 9 candidates included 8 flagellar proteins, from which FliK was selected, and the outer membrane protein A (OmpA).

The flagellum of *Campylobacter* is a crucial virulence factor that contributes to motility, chemotaxis, protein secretion and evasion of the innate immune response [23]. Despite the absence of studies on the role of FliK in *C. fetus*, studies on C. *jejuni* showed that this protein plays a role in flagellar hook-length control, similar to the well-studied homolog in *Salmonella* [21, 24]. Deletion of FliK in *C. jejuni* produced abnormally long hooks [25]. Using a reverse vaccinology approach, Li et al. [26] identified FliK as a promising vaccine candidate against *Salmonella*, and immunization of mice with this protein triggered an antibody-mediated immune response and reduced mortality. This protein potentially confers protective immunity by triggering an antibody-mediated response, which may disrupt the length of flagella, thus reducing colonization and invasion [26].

The OmpA family is a group of genetically related surface-exposed proteins present in high copy numbers, involved in pathogenicity, by mediating adhesion, cell invasion and serum resistance [27, 28]. These characteristics make OmpA proteins suitable candidates for vaccine development [28]. In fact, OmpA was identified by several studies as a promising vaccine target against other bacterial species, such as *Shigella flexneri* and *Acinetobacter baumannii*, as it stimulates a protective immune response [29, 30] and induces specific humoral and cytotoxic immune responses [27].

Memory helper T and B cells play a crucial role in triggering an immune response against future infections, providing protective immunity [17]. In the current study, T-cell epitopes binding to MHC class I and II were predicted from B-cell linear epitope sequences, and the top-ranked were used to design a multi-epitope vaccine. These epitopes were conserved across multiple *Cfv* strains. An EAAAK linker, which is a rigid α-helix linker, was used to connect the adjuvant (Cholera toxin B subunit) to the multi-epitope fragment, ensuring efficient separation from other vaccine domains [31]. Additionally, GPGPG linkers were included between epitopes to avoid the formation of junctional epitopes, by acting as spacers, which facilitates epitope processing and presentation by the immune cells [32, 33]. The resulting subunit vaccine consisted of 370 residues. Prediction of antigenicity and allergenicity provided information about the vaccine efficacy and safety. The vaccine construct exhibited non-allergenicity, high antigenicity and good solubility upon overexpression, meeting the requirements for eliciting a strong immune response [33].

Codon optimization was implemented to enhance the efficiency of transcription and translation of the recombinant vaccine in *E. coli.* This optimization allows vaccine production with high expression levels. The CAI of 1 and GC content of 50.36% are compatible with an effective expression of the vaccine construct in *E. coli* strain K12 [33].

The vaccine recipient’s immune response was simulated using the C-ImmSim server, which conducts *in silico* experiments that simulate vaccine injections at different time intervals. This immune simulation provided valuable insights into the efficacy of the vaccine in inducing both humoral and cellular immune responses. The vaccine construct was able to trigger an antibody response and promoted the production of memory cells. During the entire simulation period, IgG and IgM levels persisted although showing a gradual decrease. Previous research suggests that systemic vaccination can confer protective or therapeutic effects by stimulating the production of IgG antibodies [5]. These antibodies play a role in hindering bacterial adhesion and mobility, activating the complement system, and participating in opsonization [5].

## Conclusions

This study developed a multi-epitope vaccine candidate against *Cfv*, with potential to be used in the control of BGC. Using reverse vaccinology, the proteins FliK and OmpA, known for their significant roles in bacterial virulence and disease pathogenesis, were identified as promising vaccine targets, and the most promising epitopes of these proteins were included in the design of the multi-epitope vaccine. *In silico* immune simulations indicate a potential efficacy of this vaccine candidate. To validate this efficacy and the effectiveness of the vaccine in prevention and treatment of *C. fetus* subsp. *venerealis* infection, in vitro expression and in vivo experimental studies are now needed.

## Methods

**Fig. 6:**
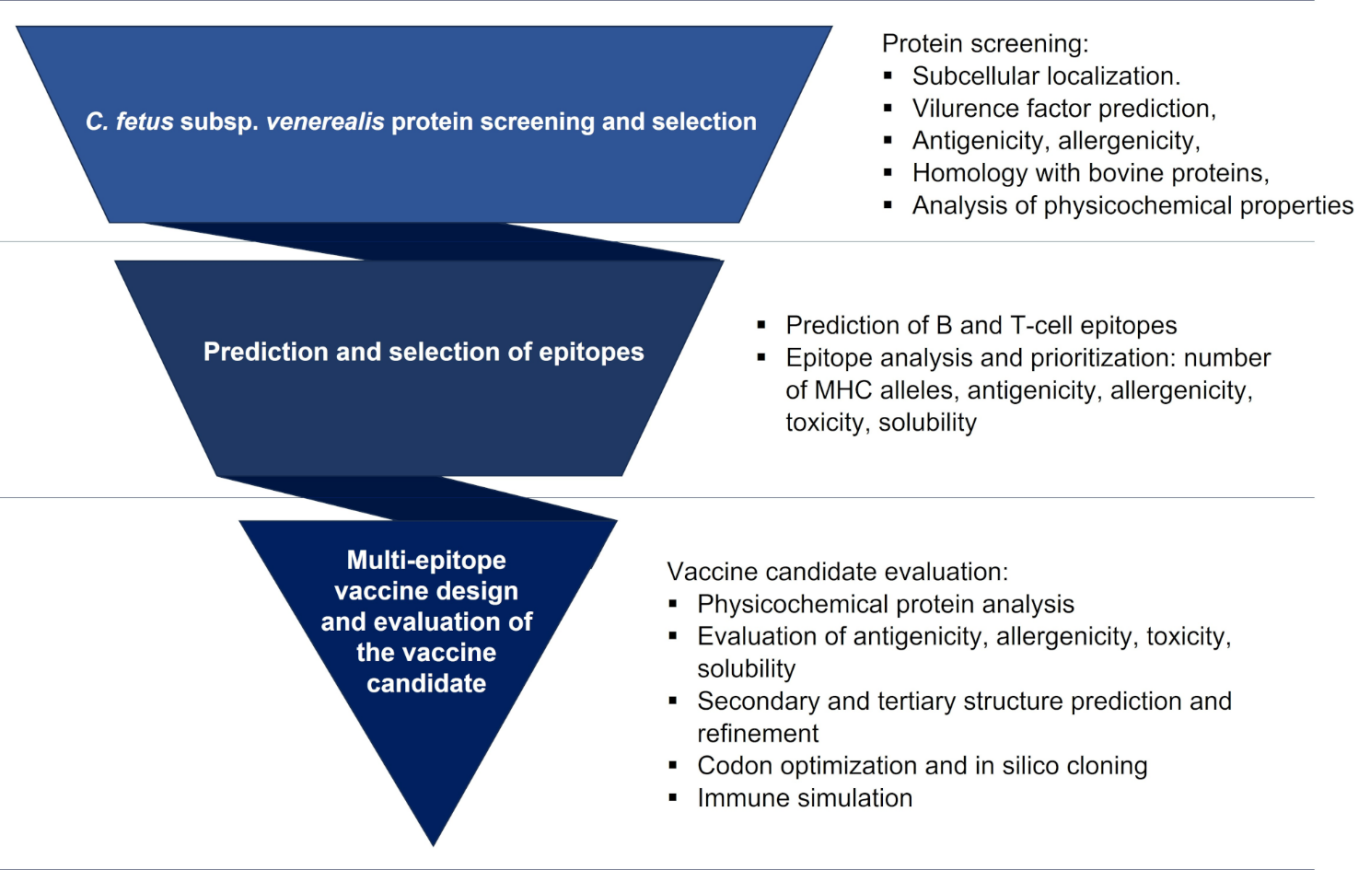
Flow-chart illustrating the methodology employed in this study for developing a multi-epitope vaccine candidate against *C. fetus* subsp. *venerealis*

This study analysed *Cfv* proteome using a reverse vaccinology approach, to identify promising candidates for the development of a multi-epitope vaccine. The methodology, described in detail in this section, is summarized in Fig. 6.

### Proteome retrieval and protein screening

The whole proteome of *Cfv* strain NCTC 10354 was obtained from the GenBank database in FASTA format (NCBI Reference Sequence: NZ_CP043435.1), and the subcellular localization of proteins was predicted using PSORTb version 3.0.3 (https://www.psort.org/psortb/) with settings for Gram-Negative bacteria [34]. Proteins putatively involved in NCTC 10354 virulence were identified using the VFanalyzer tool of the Virulence Factor Database (VFDB) (http://www.mgc.ac.cn/cgi-bin/VFs/v5/main.cgi) [35]. Only extracellular and outer membrane proteins, classified as potential virulence factors, were selected for further analysis. Surface array proteins (Sap) were excluded due to the high-frequency of *Campylobacter fetus* antigenic variants of SLPs, used to evade the host’s immune response [36].

### Antigenicity and allergenicity

Protein antigenicity was predicted using the VaxiJen version 2.0 online server (https://www.ddg-pharmfac.net/vaxijen/VaxiJen/VaxiJen.html) [37] with default settings for bacteria and a threshold value of 0.4. Prediction of allergenic proteins was performed with AlgPred version 2.0 online server (https://webs.iiitd.edu.in/raghava/algpred2/) [38] with default settings and the threshold value of 0.3. Only proteins classified as probable antigens and non-allergens were considered for subsequent analysis.

### Selection of non-homologous proteins

The exclusion of bovine homologous proteins is essential to avoid cross-reactivity of the vaccinal immune response with proteins of bovine origin. Therefore, selected proteins were analysed through PSI-BLAST (https://blast.ncbi.nlm.nih.gov/Blast.cgi?CMD=Web&PAGE=Proteins&PROGRAM=blastp&RUN_PSIBLAST=on) [39], and only non-similar proteins, with no hits found on PSI-BLAST, using a threshold of 0.005, were selected for subsequent analysis.

### Analysis of physicochemical properties and protein prioritization

The proteins were then analysed for their physicochemical properties. The number of amino acids, instability index, aliphatic index and GRAVY were assessed with the ProtParam toll of the Expasy web platform (https://web.expasy.org/protparam/) [40]. Non-stable proteins, with an instability index higher than 40, were excluded, and to select hydrophilic and thermal stable molecules, only proteins with a negative GRAVY and an aliphatic index higher than 50 were selected. Since proteins with more than one transmembrane helix are difficult to purify [41], prediction of transmembrane helices was performed with HMMTOP server version 2.0 (http://www.enzim.hu/hmmtop/index.php) [42], and proteins with more than one transmembrane helix were also excluded. Proteins fulfilling the above criteria were prioritized, and 2 of these proteins were selected for subsequent epitope analysis for antigenicity score on VaxiJen and cell function.

### Prediction of B and T-cell epitopes

Linear B-cell epitopes were predicted using the BepiPred version 2.0 from the IEDB analysis resource online platform (http://tools.iedb.org/bcell/) [43], using a threshold value of 0.5. The sequences of linear B-cell epitopes with more than 10 residues were used to predict MHC type I and type II binding peptides. Epitopes potentially immunogenic to cytotoxic T Lymphocytes (CTL) were predicted by identifying peptides binding to MHC class I molecules, using NetMHCpan version 4.1 from the DTU Health Tech platform (https://services.healthtech.dtu.dk/services/NetMHCpan-4.1/) [44]. The analysis was performed for the 105 BoLA alleles available, for identification of 9-mer peptides. Only strong binding results were considered, using the 0.5% threshold defined by default. Prediction of peptides binding to MHC class II BoLA-DRB3 alleles was performed using NetBoLAIIpan version 1.0 (https://services.healthtech.dtu.dk/services/NetBoLAIIpan-1.0/) [45]. This evaluation included BoLA-DRB3 alleles from mass spectrometry for the identification of peptides with 15 residues, and only strong binding results were considered, using the 1.0% threshold defined by default.

### Epitope analysis and prioritization

The predicted epitopes were then evaluated to confirm their potential antigenicity and non-allergenicity, using VaxiJen version 2.0 and AllerTOP version 2.0 (https://www.ddg-pharmfac.net/AllerTOP/) [46], respectively. The peptides were also confirmed to be non-toxic using ToxinPred (https://webs.iiitd.edu.in/raghava/toxinpred/) [47]. Additionally, the solubility was evaluated with the peptide solubility calculator from Innovagen (https://pepcalc.com/peptide-solubility-calculator.php) and only peptides with good water solubility remained under analysis. The epitopes meeting the above criteria were prioritized based on their ability to bind to several MHC alleles and their antigenicity score.

### Multi-epitope vaccine design

At the end of the above analyses, 15 epitopes arising from the 2 proteins were selected for a multi-epitope vaccine design. The conservation of the amino acid sequence of the epitopes was analysed using BLAST in a collection of 31 *Cfv* genomes analysed in a previous study [48]. The epitopes were then linked together by GPGPG linkers, and the multi-epitope peptide sequence was bonded to the cholera toxin B subunit sequence using an EAAAK linker. The cholera toxin B subunit was included as an adjuvant to stimulate a strong immune response.

The physicochemical properties of the vaccine construct were evaluated with the Protparam tool. Additionally, antigenicity, non-allergenicity and non-toxicity were assessed with Vaxijen version 2.0, Allertop version 2.0 and ToxinPred, respectively. The solubility upon overexpression was predicted with SolPro (https://scratch.proteomics.ics.uci.edu) [49], considering acceptable a solubility higher than 0.5. Thereafter, the secondary structure of the vaccine candidate was predicted with PSIPRED version 4.0 (http://bioinf.cs.ucl.ac.uk/psipred/) [50] and the tertiary structure was predicted using the Scratch Protein Predictor – 3Dpro (https://scratch.proteomics.ics.uci.edu/) [51]. The structure model was refined using the Galaxy Refine tool of the GalaxyWEB server (https://galaxy.seoklab.org/cgi-bin/submit.cgi?type=REFINE) [52] and was subjected to disulfide engineering with Disulfide by Design version 2.0 (http://cptweb.cpt.wayne.edu/DbD2/index.php) [53]. Disulfide bonds were added to increase structure stability in residue pairs having an energy value less than 2.2 Kcal/mol, mutating the residues to cysteine.

### Codon optimization and in silico cloning

To achieve a high expression of the vaccine candidate in *Escherichia coli*, codon optimization was performed using the Java Codon Adaptation Tool (JCat) (https://www.jcat.de/) [54] for *E. coli* K12. The CAI and GC content were used to estimate the potential expression level of the vaccine. The optimal CAI score is 1, although a score above 0.8 is considered acceptable [55]. Additionally, the GC content should ideally range between 30 and 70% [33]. The improved DNA sequence was *in silico* cloned on a vector pET-30a(+) using SnapGene® software (from Dotmatics; available at snapgene.com). The *in silico* cloning included the simulation of a restriction digestion with NotI and BamHI and the ligation of the nucleotide fragment encoding the vaccine candidate.

### Immune simulation

An immune simulation of the vaccine construct was performed with C-ImmSim server (https://kraken.iac.rm.cnr.it/C-IMMSIM/) [56] for 1000 simulation steps, with two injections. Injections included 1000 antigens, as defined by default, and were administered at 1 and 84 time steps, corresponding to days 1 and 28 since each time step represents 8 h. The default settings were used for the remaining parameters of the simulation.

### Electronic supplementary material

Below is the link to the electronic supplementary material.

Supplementary Material 1

Supplementary Material 2

Supplementary Material 3

## Data Availability

All data supporting the findings of this study are included in this paper and its additional files.
