# Supplementary figures and images for: Design of a multi-epitope-based vaccine candidate against Bovine Genital Campylobacteriosis using a reverse vaccinology approach
Source: BMC Vet Res. 2024 Apr 19;20:144. doi: 10.1186/s12917-024-04006-x (PMC11027316; doi:10.1186/s12917-024-04006-x)

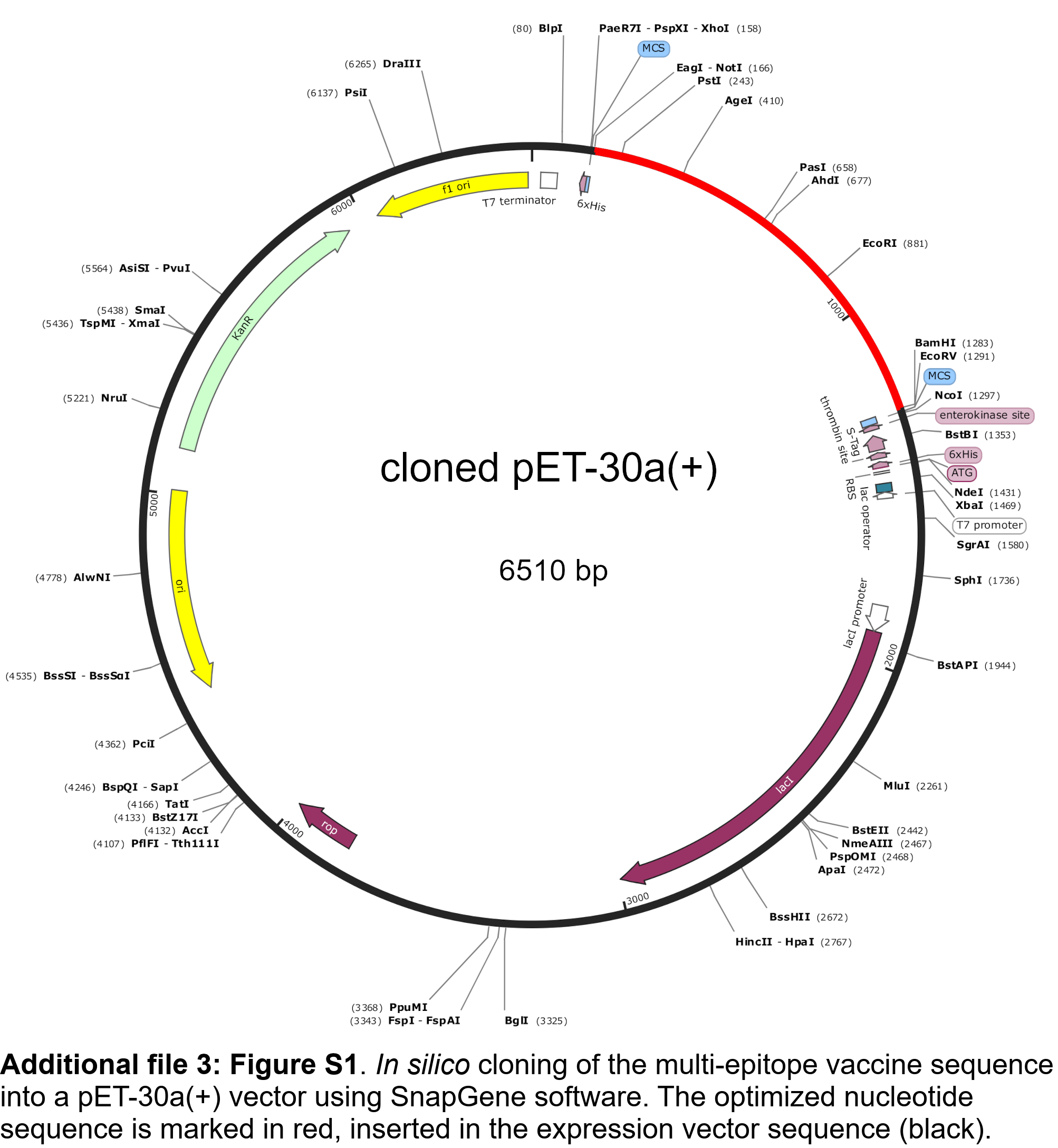

Supplement: Supplementary file 3 — Supplementary Material 3 [file 12917_2024_4006_MOESM3_ESM.tif]
